# There Is No Place Like Home: Behavioral and Physical Home Traits in Humans and Other Animals

**DOI:** 10.1093/biosci/biaf183

**Published:** 2025-12-04

**Authors:** Efrat Blumenfeld Lieberthal, David Eilam

**Affiliations:** Azrieli School of Architecture, The David and Yolanda Katz Faculty of the Arts, Tel Aviv University, Israel; School of Zoology, The George S. Wise Faculty of Life Sciences, Tel Aviv University, Israel

**Keywords:** identity, spatial behavior, personal space, living range, nomadism

## Abstract

In both humans and other animals, home demonstrates a typical functional organization, as well as behavioral and cognitive perspectives. It is an extension of individual space and accordingly reflects the identity and personification of its inhabitants. Beyond its role as a place of comfort, rest, and intimacy, home functions as a hub and anchor for traveling away from it, organizing patterns of movement, routines, and spatial memories. These, altogether, form a living range that may be considered an extension of home. Indeed, the traits of home manifest across multiple scales, ranging from the individual’s home to hometown, homeland, and, ultimately, Earth itself. Finally, the concept of home is also relevant in both homeless humans and animals that develop behavioral home substitutes under conditions of transience, mobility, or displacement. Collectively, the concept of home-at-large reveals notable similarities and a convergence of home-related behaviors between human and nonhuman species.

Home, at its core, provides protection and a secure environment. However, the concept of home extends across species, encompassing physical, symbolic, emotional, and functional dimensions. Traditionally, human homes are described in architectural, psychological, or sociological terms (Després 1991, Douglas 1991), whereas animal dwellings are perceived as mere shelters in biological or functional categories (Burns et al. 1989, Bronner 1992, Hansell 1993, Kinlaw 1999). However, a closer examination reveals striking structural similarities between the homes of humans and those of other animals, as well as the behavior associated with those homes (Edney 1976). From laboratory rodents dividing their space into functional sites (Leonard and McNaughton 1990) to polar bears constructing bifurcated dens for rearing young and sheltering (Durner et al. 2003) and beehives with nurseries and food compartments (Winston 1991), many animal species organize space in ways that resemble human domestic layouts. Furthermore, humans customize their living environments to reflect identity, security, and emotional belonging, and animals demonstrate similar behavioral patterns, such as nest decoration (Diamond 1986), spatial regularity (Gorman and Trowbridge 1989), and territory demarcation (Blank 2025), which suggest the presence of a home logic far beyond survival. In this article, we explore home not merely as a built or inherited structure but as a behavioral, cognitive, and emotional phenomenon. Drawing examples from bowerbirds (Diamond 1986), adolescent bedrooms, and astronauts in orbit (Yaden et al. 2016), we examine how home manifests across species, spaces, and scales. We argue that the broad notion of home is not exclusive to humans, nor is it reducible to shelter. Rather, it represents a shared evolutionary behavioral pattern that includes spatial anchoring (Yaski and Eilam 2007), identity expression (Porteous 1976, Wise 2000), social distinction (Hall and Hall 1966), and routine movement (Walther 1977, Gonzalez et al. 2008). These patterns occur across physical, psychological, and cultural dimensions, as well as across individual, group, and national levels. By uncovering these common characteristics, the concept of home is positioned as a universal behavioral and symbolic anchor (Couclelis et al. 1987) that organizes spatial experience in both humans and other animals.

Drawing on ethnographic case studies, vernacular architecture, and comparative animal behavior, six thematic sections follow, each exploring the multidimensional nature of home across humans and different animal species, highlighting a distinct facet of home as both a physical and behavioral phenomenon: comparing the functional spatial organization of homes in humans and animals, focusing on how both construct dedicated zones for activity, rest, and raising offspring; examining home as an extension of the individual space, drawing on the concept of personal territory and its manifestation in both humans and animal behavior; exploring the relationship between home and identity, considering how personalization and symbolic expression transform dwellings into reflections of self, in both humans and animals; analyzing home as a cognitive and spatial anchor, organizing patterns of movement, routine, and spatial memory; studying the scaling of home from individual dwellings to cities, nations, and even Earth itself, exploring how notions of belonging and identity scale up; and illustrating how both humans and animals develop behavioral substitutes for home under conditions of transience, mobility, or displacement.

Following the notion that the meaning of *home* ought to be scrutinized as a multidimensional concept (Mallett 2004), we identify and compare recurring dimensions of domestic space: functional, spatiobehavioral, affective–symbolic, sociocultural, and temporal–dynamic, because they manifest across cultural and biological contexts.

Our examples illustrate various dimensions of homeliness rather than identifying a single species that mirrors the human home in its entirety. Because human homes integrate multiple functions—structural, social, symbolic, spatial, and temporal—we draw on diverse taxa that exemplify each dimension most clearly within their ecological context. For example, subterranean**-**dwelling species such as voles, jirds, and gerbils demonstrate functional spatial partitioning within physically constrained environments, offering direct parallels to the dedicated zones of human dwellings. In contrast, large free-ranging species, such as ungulates, illustrate social and spatial homeliness at the collective level, where group organization, synchronized routines, and site fidelity compensate for the absence of permanent structures. Other taxa, including bowerbirds, social insects, and migratory birds, highlight yet further dimensions, from symbolic personalization to rhythmic seasonal reconfiguration of their homes. This comparative sampling reflects an intentional, multiscalar approach: rather than seeking one-to-one analogies, we map how distinct components of homeliness are distributed across species, revealing both shared behavioral principles and context-specific adaptations. This integrative approach highlights both common patterns and meaningful divergences in how beings create, inhabit, and interpret home. We begin with the most tangible aspect of home: the physical and functional structures that provide shelter and safety across species.

## Roof over one’s head: Home as a physical and functional structure

Home is a physical construct, in its most foundational form, a defined and bounded space that accommodates essential biological and social functions, providing a shelter and sense of safety. This applies to both humans (Coolen and Meesters 2012) and nonhuman animals (Laidre 2021). Although human dwellings are often described in architectural or design terms, animal shelters are traditionally viewed as crude or instinctive constructs. Nonetheless, a closer examination reveals remarkable structural and functional parallels between the two.

**Figure 1.**
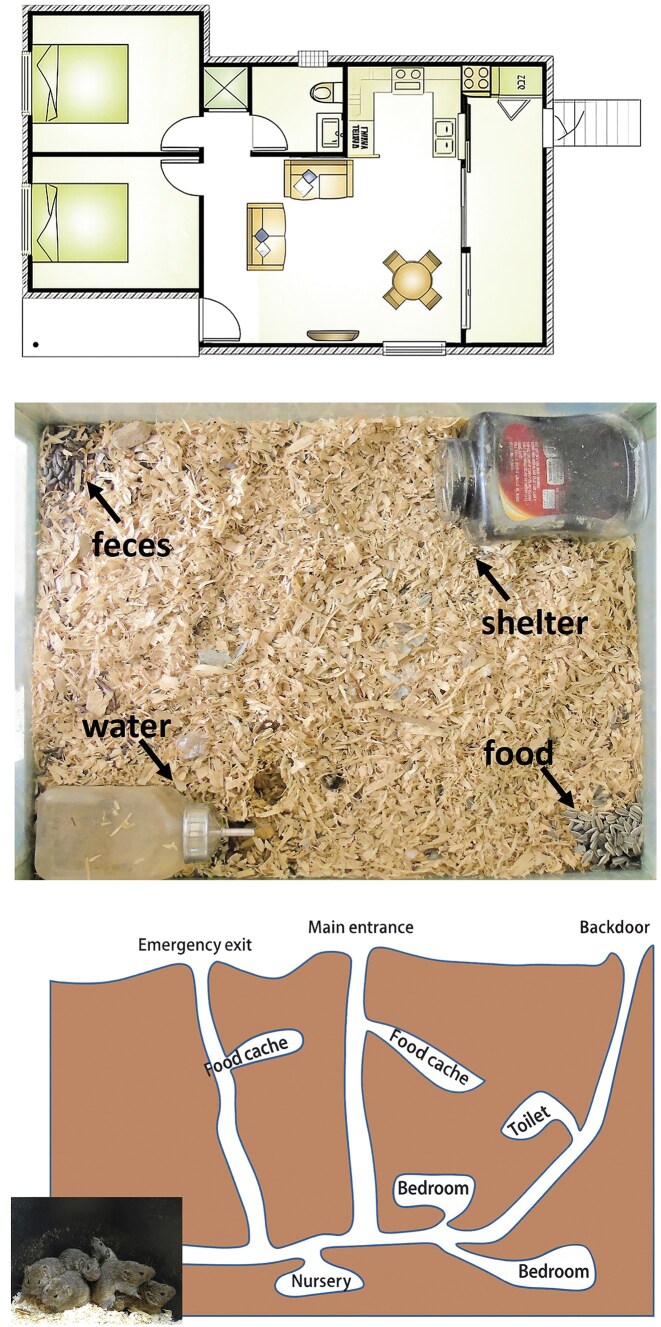
The general similarity in the functional division of dwellings in humans and animals. Top: layout of a human apartment divided to living room, bedrooms, kitchen, shower and toilet. Center: a small captive cage of a wild black rat (*Rattus rattus*), with a latrine in one corner, shelter in another, and food storage in another corner. Bottom: a sketch of a burrow system of a vole family divided to bedrooms, nursery, toilets, and food caches.

Human homes are typically divided into zones of specific activities: living rooms for social interaction and rest, kitchens for cooking, bedrooms for sleep and privacy, and bathrooms for hygiene. This spatial partitioning follows cultural norms and practical needs but is not uniquely human. Among animals, similar forms of functional zoning are evident. Laboratory rats (*Rattus norvegicus*) partition their environment in both open arenas and confined cages. When released into a large room, they created and maintained separate areas for nesting, food storage, movement corridors, and waste elimination (Leonard and McNaughton 1990), and even in small cages, they demonstrated consistent spatial preferences, indicating an innate drive for environmental structuring (figure 1). This behavior reflects an internal spatial order that optimizes comfort, hygiene, and survival.

In natural habitats, the burrow systems of various rodents such as voles, jirds, and gerbils show elaborated internal organization. These subterranean structures typically include designated chambers for sleep, nurseries for rearing young, storage rooms for hoarded food, latrine areas, escape tunnels, and ventilation holes (figure 1). Such complexity illustrates that various species exhibit purposeful spatial behavior, driven by fundamental needs for safety, cleanliness, and caregiving. Although many animals dig simple tunnels ending in a single chamber, more elaborate examples exist. For instance, the long-eared hedgehog (*Hemiechinus auritus*) and the polar bear (*Ursus maritimus*) construct dens with bifurcated endings: two separate chambers, one for the offspring and one for the mother. This spatial division supports concurrent caregiving and rest, reinforcing the idea that even solitary animals engage in sophisticated spatial logic when it comes to rearing young (Mendelssohn and Yom-Tov 1999, Durner et al. 2003).

**Figure 2.**
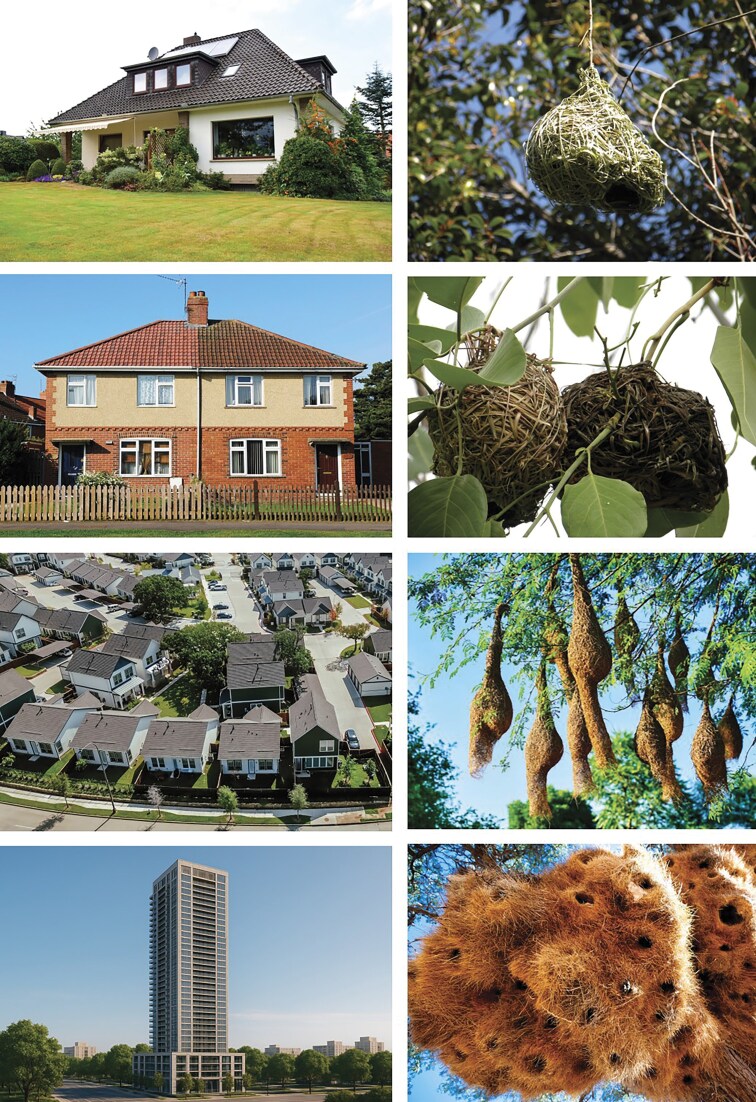
A parallel between human homes (left) and nests of various species of weaver birds (right), both range from an isolated house, via a semidetached, a neighborhood, up to a communal residence.

Just as humans live in detached houses, semidetached units, or apartments, animals vary in their physical proximity to conspecifics. Some, such as the blind mole rat (*Spalax ehrenbergi*), are fiercely solitary, maintaining and defending isolated burrow systems (Zuri and Terkel 1996). Others, such as the fat sand rat (*Psammomys obesus*), may live individually but build burrow networks in close proximity to others (Ilan and Yom-Tov 1990)‏. Still others, such as the social Guenther’s vole (*Microtus guentheri*), live in extended familial groups, constructing complex communal networks of interconnected burrows with shared access points and storage chambers (Cohen-Shlagman 1981). These patterns mirror the human condition, where residential environments vary from isolated rural homes to tightly packed urban apartments (figure 2). However, the same imperative shapes all to organize space in relation to individual and social needs.

These observations suggest that the organization of domestic space follows a set of underlying principles that transcend species. Whether shaped by architectural intention or biological instinct, homes across humans and animals tend to include zones for rest, protection, resource management, reproduction, and social interaction. Despite the vast differences in scale, material, and cognitive capacity, the logic underlying the home’s spatial structure appears to be deeply rooted in functional necessity and behavioral regularity. This convergence suggests that homemaking is not merely a cultural act but a universal behavior with shared architectural motifs and biological needs.

Although the structure of a home reflects basic biological needs, its meaning extends beyond functionality. Home is not only a collection of spatial zones but is deeply intertwined with self-perception and boundaries. As animals and humans divide space for practical purposes, they also assign psychological significance, creating zones of proximity and protection that mirror social and emotional hierarchies. Therefore, home extends personal space shaped not just by survival needs but by cognitive, emotional, and social factors. The next section examines how home functions as a psychological perimeter, regulating interpersonal distance, defining identity and privacy, and reflecting behavioral patterns across species.

## My home is my castle: Home as an extension of personal space

Although the physical layout of a home reflects practical considerations, it also encodes subtle psychological boundaries that shape how individuals experience proximity, interaction, and identity. These boundaries resonate with Edward T. Hall’s (Hall and Hall 1966) theory of proxemics, which introduced the concept of personal space as an invisible, culturally conditioned zone surrounding the individual. Hall identified multiple concentric zones: intimate, personal, social, and public, each regulating levels of comfort and perceived intrusion. These distinctions are not just social abstractions; they manifest in the organization of domestic space.

A loose parallel between these concentric zones and the organization of domestic space can be drawn. Within the home, rooms often align with different degrees of access and relational closeness: Living rooms serve as social zones, open to guests and shared activities; bedrooms provide personal and intimate spaces, reserved for close relations, whereas bathrooms or private corners represent heightened privacy and vulnerability. Although they are not measured in centimeters, these spatial divisions reflect a similar logic of exposure and control, defining who may enter which zones and under what circumstances.

Notably, this layered spatial logic is not exclusive to humans. Many animals also regulate proximity and defend boundaries. For instance, birds roosting on a branch maintain what has been described as pecking distance, a minimum space that prevents aggression and ensures individual comfort (Keeling 1994, Côté 2000, Fernández-Juricic et al. 2007). Similarly, territorial animals, ranging from fish to mammals, display escalating levels of aggression as intruders approach the core of their territory.

**Figure 3.**
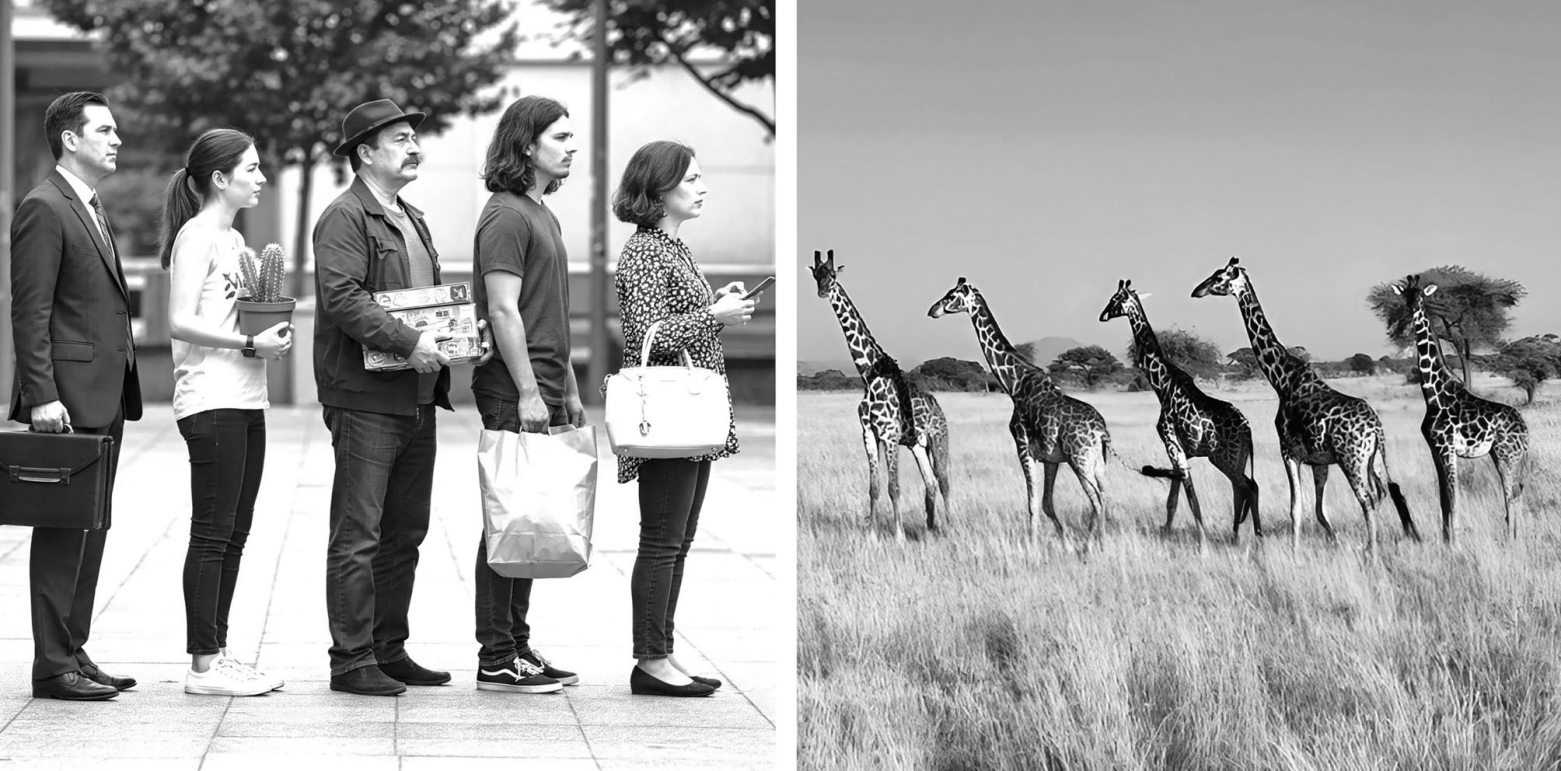
Personal space and spacing behavior in humans and animals. A comparison between the distancing maintained by individuals in a human queue and the spacing between individuals in a Masai giraffe (*Giraffa tippelskirchi*) caravan. Both illustrate an innate regulation of personal space that balances proximity with individual comfort and safety.

Similar behavioral dynamics in humans are evident in public and domestic settings. People queuing in line instinctively maintain personal space from others, and a violation of this invisible boundary often triggers discomfort or subtle avoidance cues (figure 3; Furnham et al. 2020). Inside a home, these spatial rules become even more finely tuned. Who is allowed into the house and how far they may penetrate it are governed by social codes, cultural norms, and psychological comfort. The threshold, such as the front door, is a critical marker: a point at which the outside world must seek permission to enter. Once inside, the progression through increasingly intimate spaces is carefully managed.

This territorial sensitivity is often reinforced through spatial personalization and symbolic boundary making. Just as many mammals mark their environment with scent or visual cues, humans use design, décor, and object placement to delineate ownership and establish a sense of control. Personalization is not merely decorative; it is a behavioral signal that states, “This space is mine.”

The psychological significance of this spatial personalization becomes clear when it is disrupted. Burglary or unauthorized entry often trigger intense emotional reactions: anger, fear, and a sense of violation (Brown and Harris 1989, Douglas 1991). These reactions are not solely responses to material loss; they are expressions of boundary violation that is also experienced under voyeurism. In this sense, the home is experienced as an extension of the self, and its violation feels akin to a personal assault.

Similar patterns of escalating defense and negotiated boundary setting also appear in nonhuman species. Lorenz (1974) describes how a three-spined stickleback fish (*Gasterosteus aculeatus*) increases aggression as an intruder approaches its territorial core. As the intruder retreats, the role is reversed: The original attacker, now farther from its core, reduces aggression, whereas the other fish, defending its center, becomes more assertive. This pendulum-like cycle of escalation and retreat continues until equilibrium is reached, switching to threat displays instead of attacks. This stabilized boundary, formed through iterative interaction, effectively marks the negotiated border between respective territories. Indeed, iterative marking of territory borders by, for example, olfactory cues in red foxes (*Vulpes vulpes*; Giuggioli et al. 2011) is a salient behavior in many animal species.

These patterns, across species, settings, and levels of consciousness, reveal a shared behavioral logic: Home operates as an expanded perimeter of the self, offering a spatial framework for negotiating one’s place in the world. Whether marked by scent, decoration, ritual, or aggression, this space is more than a shelter; it is a territory made meaningful and shaped not only by architecture or instinct but by cognition, emotion, and culture. Personal space regulates access and proximity, but personalization and symbolic expression transform a house into a home.

## Make a house home: Identity, ownership, and the sense of belonging

Beyond its structural and territorial functions, home serves as a profound symbol of identity and belonging. It is a reflection of the self, expressing who we are, where we come from, and sometimes, who we wish to become. It is where we can be ourselves, express our individuality, and feel comfortable. The way we decorate, arrange furniture, and choose colors reflects our personal tastes, values, and experiences and creates a space where we can curate our surroundings to create an environment that resonates with our sense of self (Wise 2000). Porteous (1976) proposed that the notion of home extends beyond its physical structure into three key social and individual dimensions: identity, security, and stimulation. This framework helps to illuminate why the expressive role of home is so deeply embedded across human cultures, life stages, and animal behavior, suggesting that the impulse to inscribe meaning onto space is both universal and evolutionarily grounded.

**Figure 4.**
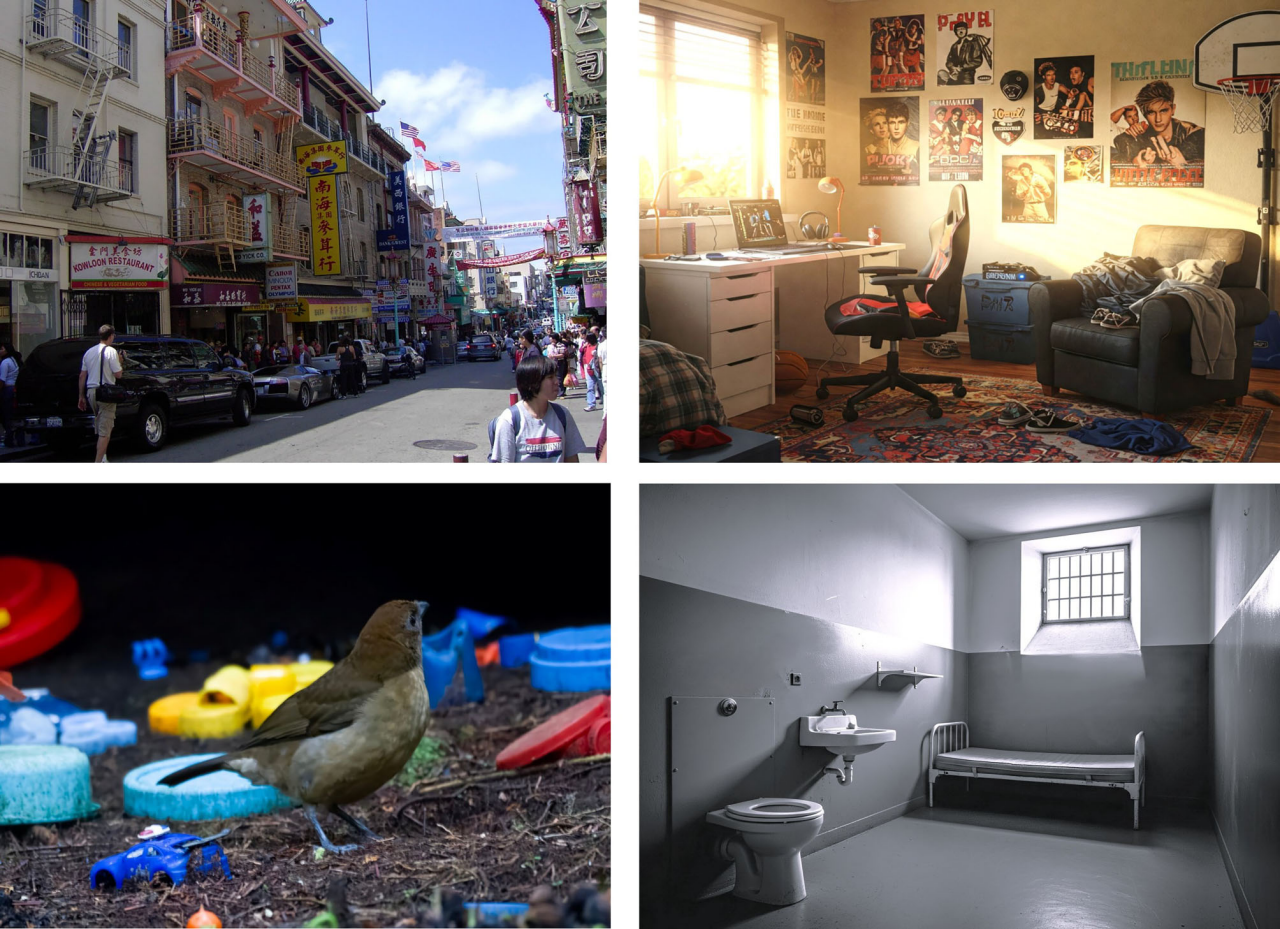
Expressions of identity and cultural meaning in personal spaces. Top left: San Francisco’s Chinatown, where collective identity is expressed in the built environment. Bottom left: a Vogelkop bowerbird (*Amblyornis inornata*) nest decorated with objects to attract mates. Top right: a teenager’s room, personalized with objects reflecting individual identity and preferences. Bottom right: a prison cell, where personal expression is constrained.

Among adolescents, bedrooms often serve as both a retreat and a stage for self-definition. Teenagers frequently modify their rooms with posters, photos, colors, lighting, and furniture arrangements that reflect their emotional states, interests, and aspirations. Such personalization transforms neutral architecture into a site of identity expression, demarcating the adolescent’s space as their own, a first step toward independence. In effect, the room becomes a three-dimensional self-portrait, echoing Jung’s view of home as a symbolic representation of the self, where design choices reflect self-perceptions and deeper psychological needs. This behavior mirrors that of the males in many bird species, where they construct and sometimes decorate their nest to display their quality as mates. For example, males of the Vogelkop bowerbird (*Amblyornis inornata*) build a bower decorated with sticks and bright objects in an attempt to attract a mate (figure 4; Diamond 1986). Like the adolescent room, the bower is an external manifestation of identity, communicating fitness, taste, and distinctiveness to the outside world, a version of courtship display or coloration in other bird species. Altogether, personalization contributes to the sense of identity and reinforces the connection to physical home.

In humans, expressing identity through space extends far beyond the individual. Cultural groups often imprint collective identity on domestic environments. Diasporic communities such as Chinatowns across Western cities illustrate this well: Through food, furnishings, spatial layout, and decorations, migrants recreate familiar environments that anchor their identity in unfamiliar settings (figure 4). Broader cultural norms also shape domestic space, as is seen in the contrasting layouts of Muslim and Western dwellings, which reflect differing social dynamics and conceptions of privacy, gender roles, and hospitality (Sebba 1991). As Zali Gurevitch (Gurevitch 2007) noted, this tendency reflects a deeper psychological process. Gurevitch recounted how Robinson Crusoe, cast away on a deserted island, instinctively rebuilt the symbolic world of home by collecting, organizing, and repurposing salvaged materials into a new domestic space. This was not mere survival but an effort to maintain cultural continuity and reaffirmed identity in a dislocated world.

Spaces stripped of personal control often produce a sense of identity erosion. Institutions such as prisons, military bases, or emergency shelters do not allow ownership. The imposed uniformity of these environments: identical beds, uniforms, communal spaces, and restrictions on personalization, which altogether reflect functional purpose and emotional emptiness (figure 4). The denial of personal space and individual boundaries undermines comfort, as well as identity (Hall and Hall 1966). In line with the concept of placelessness (Relph 1976), such environments lack the sense of rootedness, authenticity, and emotional depth that characterize meaningful places. These are not homes but spaces of containment, lacking the layers of meaning and personal imprint that define a lived-in environment. Although the impulse to personalize and control space is widespread, the way home is structured, experienced, and symbolized differs significantly across cultures. These differences highlight that home not merely as a biological or psychological concept, but also as a culturally embedded phenomenon.

In traditional Japanese architecture, for example, the home emphasizes impermanence and flexibility. Sliding doors (fusuma), tatami flooring, and multipurpose rooms allow a fluid transformation of space in response to changing needs. This reflects broader cultural values of modesty, transience, and harmony with nature (Engel 1964). In contrast, many Middle Eastern homes maintain strict internal divisions to separate gendered spaces and uphold cultural codes of privacy, honor, and hospitality. These architectural layouts are not merely functional but carry deep symbolic and social meanings.

Even in contexts of mobility, cultural traditions shape how homes are created and sustained. In nomadic cultures, the tent serves not only as a portable shelter but as a structured, symbolically rich domestic environment. The Mongolian ger, for example, is carefully oriented to face south, with interior divisions that reflect spiritual beliefs, kinship roles, and gendered tasks (Fernandez-Gimenez 2000, Humphrey 2023). Similarly, the Sámi lavvu, used by Indigenous reindeer herders in northern Scandinavia, is organized around a central hearth, facilitating social coordination and seasonal mobility (Beach 2001). Bedouin tents made of woven goat hair likewise reflect tribal structures and seasonal rhythms, with gendered divisions of space (Saidel 2009). These dwellings demonstrate that, in nomadic life, home is sustained through spatial logic, routine, and identity, not permanence.

In Western contexts, the mobility patterns of home often include the use of vacation homes or seasonal migration for retirement, such as relocating to warmer climates during winter months. These patterns reflect a culturally sanctioned form of cyclical home redefinition, emphasizing comfort, climate adaptation, and lifestyle continuity. Studies on British and Swedish retirees migrating seasonally to Mediterranean or southern European locales highlight how these individuals construct hybrid identities and establish new social routines that blur the boundaries between tourist and resident (Gustafson 2002, King et al. 2021). Similar trends are observed among US and Canadian snowbirds, humans who spend winters in states such as Arizona or Florida, often maintaining dual residences and navigating distinct health, legal, and cultural systems in the process (Stallmann and Espinoza 1996, Pickering et al. 2020). These lifestyle migrations illustrate how home can be flexibly reconfigured across time and geography, grounded less in permanence and more in rhythmic, seasonal occupation. Different seasonal socializing characterizes migrating mammals, birds and insects. During the summer, monogamous pairs of white storks (*Ciconia ciconia*) nest in permanent locations in Europe. After rearing their offspring, pairs separate, aggregate in large flocks and migrate to Africa where they spend the wintertime. By springtime, flocks return to Europe, and the same monogamous pairs meet again in their permanent location to breed and rear another generation (Leshem and Yom-Tov 1996). In another continent, wandering albatrosses (*Diomedea exulans*) can follow the same routine for 40 years or more: Monogamous pairs nest together and then separate for months until the next breeding season (Sun et al. 2022).

These diverse examples challenge the assumption that home universally reflects the same spatial or psychological logic. Instead, they show that the cultural context profoundly shapes the way space is divided, personalized, and imbued with meaning. This reinforces the view that home is not just a shelter or cognitive anchor but a cultural artefact, constructed, inhabited, and interpreted within specific symbolic and social systems. But what happens when home is disrupted or absent?

## Home away from home: Homelessness

**Figure 5.**
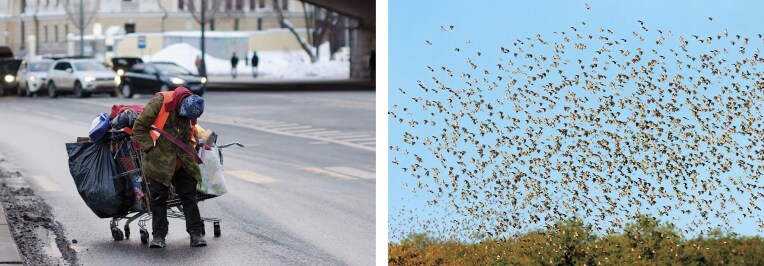
Contrasts in the experience of home. Left: human homelessness, reflecting the absence of a stable physical home despite social belonging. Right: the flock as a mobile home for red-billed quelea (*Quelea quelea*), where group cohesion provides safety and a sense of belonging in the absence of a fixed nest or territory.

The distinction between habitation and home, where *home* is detached from the physical residence (Jenkins and Brownlee 2022) is illustrated by the experience of homeless individuals who carry and carefully arrange their personal belongings often within a shopping cart (figure 5). Although this behavior may seem like the self-contained homes of turtles and snails, it actually represents efforts to recreate a sense of order, routine, and personal identity within displacement conditions. The cart of a homeless person serves as a crucial element of storage, transport, and security (Hill and Stamey 1990). The accumulation of possessions, often symbolic of a past or future identity, is common among homeless individuals, both street dwellers and shelter residents, to maintain self-esteem (Belk 1985). This psychological aspect is underscored by US federal court rulings protecting their rights to retain their belongings, highlighting the significance of possessions in forming identity, akin to home decoration in traditional dwellings. Indeed, there is a call to look at the whole life of a homeless person, rather than just at selected episodes of rooflessness (Somerville 2013). Hill and Stamey (Hill and Stamey 1990) observed that, for street dwellers, pride and identity are often derived from constructing their own living spaces, reflecting low expectations regarding conventional housing. Shelter residents, on the other hand, while receiving basic security and protection, experience a loss of control and privacy, hindering the development of a home in behavioral and emotional terms (Hill 1991). These findings support the view that home extends beyond physical structure to encompass a state of mind and associated behaviors. However, this interpretation is not universal. In Australia, homeless individuals strongly associate home with a physical dwelling, viewing it as both a solution to their homelessness and a symbol of social integration (Parsell 2012). Similarly, among nomadic communities, the social group provides the framework for belonging and coherence, allowing mobility to function as a stable home form. This social home is reflected in nomadic human groups such as Romany (Roma) and Bedouins, highlighting cultural and religious identity.

The social group can also serve as a mobile home for gregarious animals (e.g., flocks, herds, swarms, schools). In many herbivorous herds, such as those of impala (*Aepyceros melampus*), individuals merely benefit from safety in numbers: The larger the group, the lower the required level of individual vigilance and the greater the collective vigilance (Elgar 1989, Bednekoff and Lima 1998, Szulkin et al. 2006). Other herds such as those of the African buffalo (*Syncerus caffer*) display labor division with experienced individuals leading as pathfinders, whereas cows and their calves remain in the center of the herd, surrounded by fierce bulls that occupy the perimeter (Molszewski 1983). The remarkable synchrony within these herds is evident in their coordinated behaviors, including resting, feeding, locomotion, grooming, dustbathing, nursing, and elimination, all occurring on a unified schedule (Estes 1991). This coordinated behavior can extend across groups, leading to large-scale movements, as is observed in the vast migrations of African buffalo and blue wildebeest (*Connochaetes taurinus*; Molszewski 1983, Estes 1991).

The feeling of safety and comfort rendered by the group can supplant the need for a traditional physical home, particularly in nomadic social species. The red-billed quelea (*Quelea quelea*), with its immense populations across sub-Saharan Africa (about 10 billion individuals; figure 5), exemplifies this by traveling in large flocks, following resource availability (Dallimer and Jones 2002). This nomadic pattern is also seen in the cedar waxwing birds (*Bombycilla cedrorum*) in North America (Putnam 1949) and in the black swan (*Cygnus atratus*) in Australia (Kingsford et al. 1999). Insect swarms, such as migratory locusts (*Locusta migratoria*; Lecoq 2022) and army ants (*Eciton burchellii*; Willson et al. 2011) also display this behavior of following resource availability. The consistent factor across these species is their strong social nature, suggesting that the group provides a home sensation and security, compensating for the lack of a fixed location. Therefore, although the meaning of home may vary widely across cultural, environmental, and species contexts, home-related behaviors, rooted in identity, routine, and social belonging, can be observed in both humans and animals. Even in the absence of fixed or permanent dwellings, both humans and animals maintain behavioral patterns tied to orientation and return. This reveals the next dimension of home: its function as a spatial anchor.

## Home base and home runs: Home as a spatial anchor

**Figure 6.**
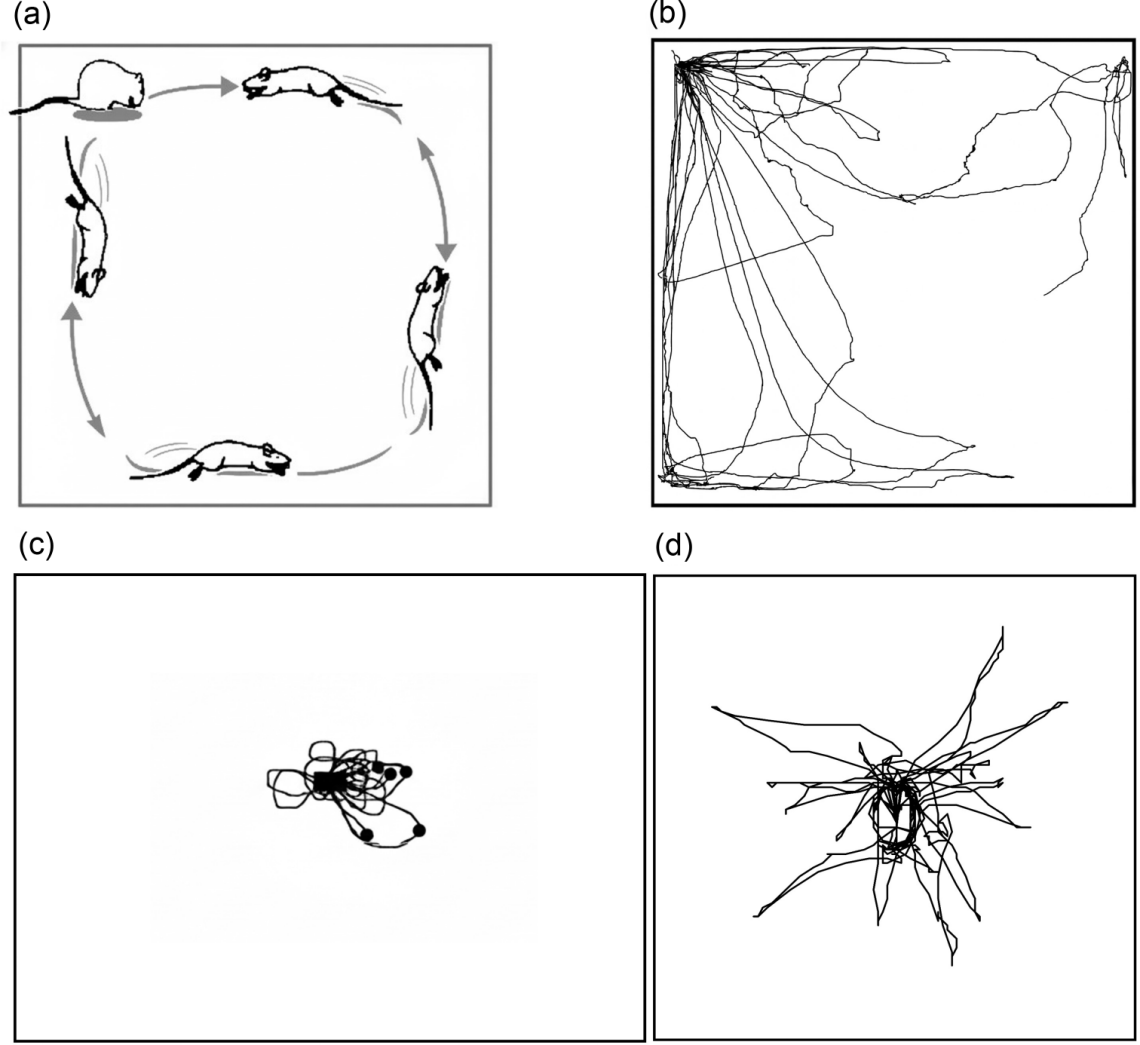
(a) An illustration of a rat that sets a home base at the top left corner where it sits and grooms and takes a round trip of exploring the circumference of the arena from there. (b) Actual trajectories of a Tristram’s jird (*Meriones tristrami*) in a 2 × 2 meter arena, with all roundtrips anchored at the top left corner: the home base. (c) Actual trajectories of a rat that was placed with a shelter in a lit parking lot (reproduced from Whishaw et al. 2006). (d) Actual trajectories of a social vole (*Microtus socialis*) that was placed in a shelter at the center of a clear arena with walls

Beyond its symbolic and emotional functions, home serves as a cognitive and spatial anchor, a reference point that shapes movement patterns and spatial behavior across species. In many cases, home functions as a terminal from which an individual departs and to which it returns, creating round-trip trajectories that reflect both environmental knowledge and predictability. When placed in an unfamiliar environment, rodents soon establish a home base, marked by characteristic behaviors, including prolonged stays, crouching, frequent returns, grooming, and rearing on the hind legs (Eilam and Golani 1989). Once a home base is established, it becomes an anchor for activity, a reference for their spatial behavior, and the rodents become engaged in structured roundtrips to the home base (home runs). These roundtrips are highly organized: The outbound part of the trip is typically slow, winding, and frequently interrupted by stops, whereas the inbound part is fast and direct (Golani et al. 1993). This pattern suggests that exploration is cognitively structured as a series of home-base-anchored trips. Indeed, the home base serves as the central organizing point of both spatial and temporal behavior (figure 6; Eilam and Golani 1989, Hines and Whishaw 2005, Nemati and Whishaw 2007, Yaski and Eilam 2007, Zadicario et al. 2007, Eilam 2010). Crouching and grooming at the home base are behaviors typical to relaxation in rodents, and altogether, these patterns indicate that home-base behavior is an innate component of spatiotemporal organization, not dependent on external structural cues. The comforting effect of the home is evident in both humans and animals, and this behavioral anchoring is what transforms otherwise identical physical spaces (e.g., identical apartments in a building or one arena corner in rat experimentation setting) into a personalized home.

In humans, like for rodents, home functions as a hub of spatiotemporal behavior, with over 50% of daily trips in humans being home generated (Golledge 1999, p. 26). Although the home anchors movement, the broader living range reveals how behaviors are distributed across space with remarkable consistency. We now turn to how specific locations within that range are behaviorally encoded.

## Home on the range: Typical behavior in specific locations of a living range

**Figure 7.**
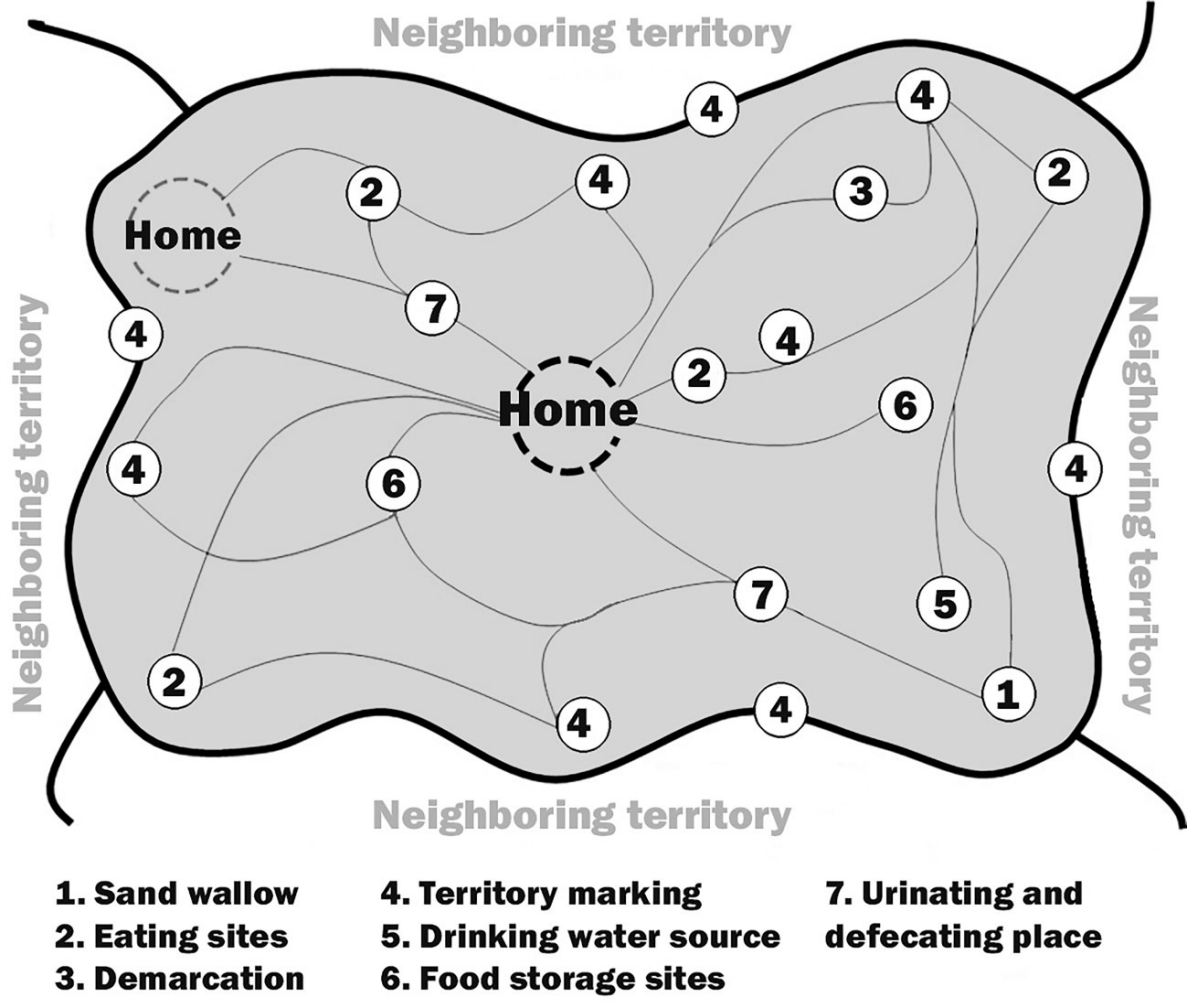
A sketch of a territory. It is centered around a home location and constitutes a set of locations, each with a typical behavior, and a network of routes that connect these locations. Source: Resketched as a follow-up of Hediger (1964).

Building on the notion of home as a cognitive anchor (Couclelis et al. 1987), it becomes clear that spatial behavior is not only centered around home but also structured through highly regular paths and routines that connect the home to key resource sites, revealing a broader pattern of route fidelity and place-specific actions across species (figure 7; Hediger 1964). In both humans and animals, repeated activity within a specific, bounded area constitutes a home range (Barrows 1996), where most actions occur (Taylor and Brower 1985, Powell 2000). Possessing a home range is thought to improve efficiency in resource use. Altogether, the spatial area where an individual typically lives and moves during routine activities is known as a home range or living range. This is the space in which daily functions such as foraging, mating, caring for young, and in humans also working and socializing occur (Edney 1976). A home range may be defended or shared with conspecifics. The size of a home range may vary significantly according to various factors, including interspecific and intraspecific interactions, resource availability, topography, physiology, and social status. For example, studies of coral reef fish show a positive correlation between body size and home range size (Kramer and Chapman 1999). However, daily movement patterns among fish are highly heterogeneous: Although some species engage in daily commutes between foraging and reproductive or resting sites, others exhibit a relatively sedentary lifestyle (Kramer and Chapman 1999). Three main movement strategies have been identified in fishes: commuting, involving crepuscular shifts between habitats; foraying, characterized by the consistent occupation of a refuge site; and mixed strategies, which incorporate features of both commuting and foraying—for example, in two species of unicornfish *Naso unicornis* and *Naso lituratus*; Meyer and Holland 2005, Marshell et al. 2011).

**Figure 8.**
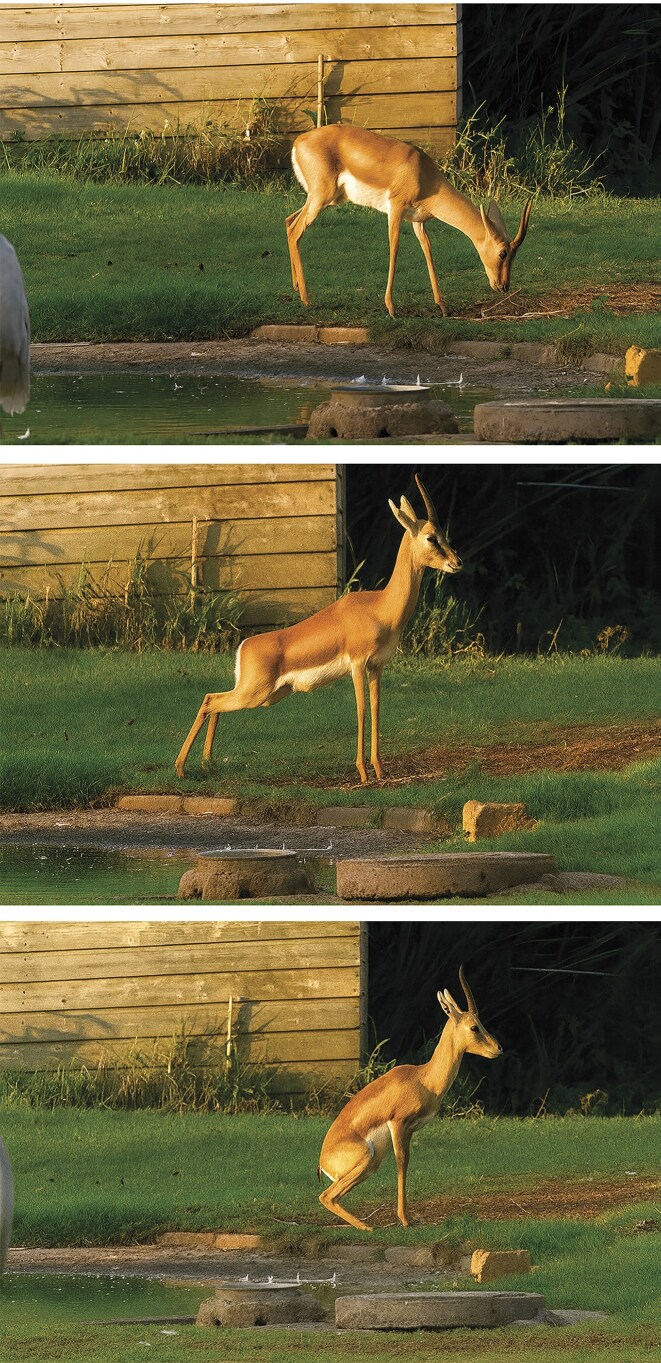
Demarcation of home territory in a captive mountain gazelle (*Gazella gazella*). Sequential phases of the ritual are shown, illustrating how physical behaviors mark and maintain a defined home range. Source: Reproduced from Eilam (2023).

The consistent performance of specific activities in designated locations is a fundamental aspect of human behavior. We typically cook and sleep at home, work in designated offices, and socialize in cafes or public venues. This tendency to associate functions with particular places reflects a deeply rooted spatial logic that extends beyond humans. Many animal species also exhibit spatial regularity, particularly in the use of marked locations for communication and territorial behavior. For instance, spotted hyenas (*Crocuta crocuta*; Gorman and Trowbridge 1989), Cape clawless otters (*Aonyx capensis*; Rowe-Rowe 1992), black rhinos (*Diceros bicornis*; Schenkel and Schenkel-Hulliger 1969), and goitered gazelles (*Gazella subgutturosa*; Blank 2025) establish specific demarcation sites within their living ranges. Antelopes display a stereotyped sequence at such sites: sniffing while scraping the ground with a forelimb, then they step forward with only the forelegs while the hind limbs stay rooted in place so that the trunk is stretched and they can urinate on the location they just sniffed. They then step forward with only the hindlegs while the forelegs are rooted in place so that the trunk is arched, allowing to defecate on the location they just sniffed and urinate on (figure 8; Eilam 2023). This repeated ritual was originally described by Walther suggesting that it is prevalent in many antelope species and produces visible territorial landmarks over time (Walther 1977). Indeed, the global generality of this ritual is reflected in its occurrence in mideast species (mountain gazelle, *Gazella gazella*; figure 8), and in an American species (pronghorn, *Antilocapra americana;* Walther 1977).

A territorial regularity of coupling between specific activities and locales is also seen in rock hyraxes (*Procavia capensis*) in the Judean Desert. Although these midsize mammals occur from the Cape of Good Hope, in Africa, all the way to the Golan Heights, in Israel, individuals inhabit a relatively small living range in which they repeatedly return to a small number of specific rocks for sitting, crouching, and scanning. In over 2000 observations, 50% of hyrax activity was concentrated on just eight rocks out of thousands available in the immediate vicinity of their shelter (Serruya and Eilam 1996). Notably, leopards (*Panthera pardus*) hunting in the same region appeared to know the hyraxes' site fidelity, occasionally executing blind jumps onto frequently used rocks without knowing whether hyraxes are there, suggesting that the leopard was familiar with the spatial regularities of the hyraxes.

The tendency to assign behavioral significance to specific locales is observable in wildlife and in human–animal parallels. An example of spatial behavior as a marker of social bonding can be seen in the monogamous golden jackal (*Canis aureus*). In this species, one behavioral indicator of a permanent pair bond is that both male and female urinate at the same places. This ritualized convergence on a specific location reflects a broader tendency among social animals to use space not only for functional purposes but also to signal affiliation and unity. This pattern resonates with human behavior, where cohabitation often involves similarly shared routines in private spaces. Spatial rituals, ranging from territorial marking to shared domestic habits, can therefore serve as indicators of relational closeness across species. Indeed, when it comes to home and its associated behaviors, humans and animals alike maintain personal space, an invisible but strongly felt boundary around the self. Entry into this space is tightly regulated and varies by species, individual temperament, familiarity, and context. Whether in territorial marking, site fidelity, or the symbolic sharing of space, these patterns underscore a universal behavioral principle: space is not neutral, it is imbued with meaning through repeated, purposeful interaction.

Two salient features commonly characterize travelling within the living range: the tendency to follow habitual routes and the recurrence of specific behaviors at key locations along these routes. The tendency to regularly travel along the same paths is a renowned trait of animals. Charles Darwin’s observations of the Galápagos giant tortoises (*Chelonoidis niger*) revealed their methodical travel patterns and location-specific behaviors, indicating a well-defined living range: “Many of the springs were a curious spectacle, to behold many of these creatures, one set eagerly travelling onward with outstretched necks, and another returning after having drunk their fill.” In addition to observing repeated use of the same paths and directionally distinct behaviors along outbound and return trips, Darwin also noted consistent behavior at the destination itself: “When the tortoise arrives at the spring, quite regardless of any spectator, he buries his head in the water above his eyes, and greedily swallows great mouthfuls, at the rate of about ten in a minute” (Darwin 1839).

These tendencies were also apparent for one of the founders of ethology, Nobel laureate Konrad Lorenz, who kept in his backyard an enclosure with water shrews (*Neomys fodiens*), a 10 centimeter energetic insectivorous mammal that lives close to fresh water, where it hunts for aquatic prey. Lorenz noticed that these shrews travel along a regular path and repeatedly jump over a specific stone along their familiar path. Astonishingly, when Lorenz removed the stone, the shrews continued to jump in that location time and again, as if unable to accept the absence of the stone. Lorenz summarized this incident by stating that “Once the shrew is well settled in its path-habits, it is strictly bound to them as a railway engine to its tracks” (Lorenz 1952). Indeed, animal pathways to resources such as water holes are often strikingly visible because of their heavy use, as is exemplified by the well-worn trails created by black rhinos (Schenkel and Schenkel-Hulliger 1969). Repeated travel along established routes relies on both internal cues (e.g., vestibular, odometric) and external environmental signals. These external cues can be directional, such as chemical, magnetic, or light gradients, providing a polarized navigation environment, especially in aquatic or low-visibility settings. Alternatively, positional cues, like landmarks, aid in determining distances and directions. Both types of cues enable consistent route following (Jacobs and Schenk 2003). For instance, salmon navigate vast, visually uniform oceans to their spawning grounds using chemical and magnetic signals, representing directional cues. Conversely, coral reef fish, such as various species of butterfly fishes (family Chaetodontidae), rely on positional cues, specifically learned coral head shapes, for foraging within their territories (Reese 1989). Even when displaced, these fish resume their established routes on encountering familiar landmarks, highlighting the importance of positional cues. The prevalence of regular route travel extends beyond fish, as is demonstrated in primates. Baboons (*Papio hamadryas*) exhibit consistent daily travel between sleeping cliffs, using fixed turning points near landmarks (Noser and Byrne 2014, Schreier and Grove 2014). Brown-mantled tamarin monkeys (*Saguinus fuscicollis*) navigate using near-to-goal landmarks (Garber and Porter 2014), and bearded sakis (*Chiropotes sagulatus*) optimize their foraging routes by memorizing high-quality food patch locations (Shaffer 2014). Furthermore, spider monkeys (*Ateles belzebuth*) and woolly monkeys (*Lagothrix poeppigii*) consistently follow stable routes within their home ranges, guided by recognizable landmarks, over extended periods (Di Fiore and Suarez 2007). This ubiquitous behavior underscores the tendency of regular travelling paths across diverse species.

**Figure 9.**
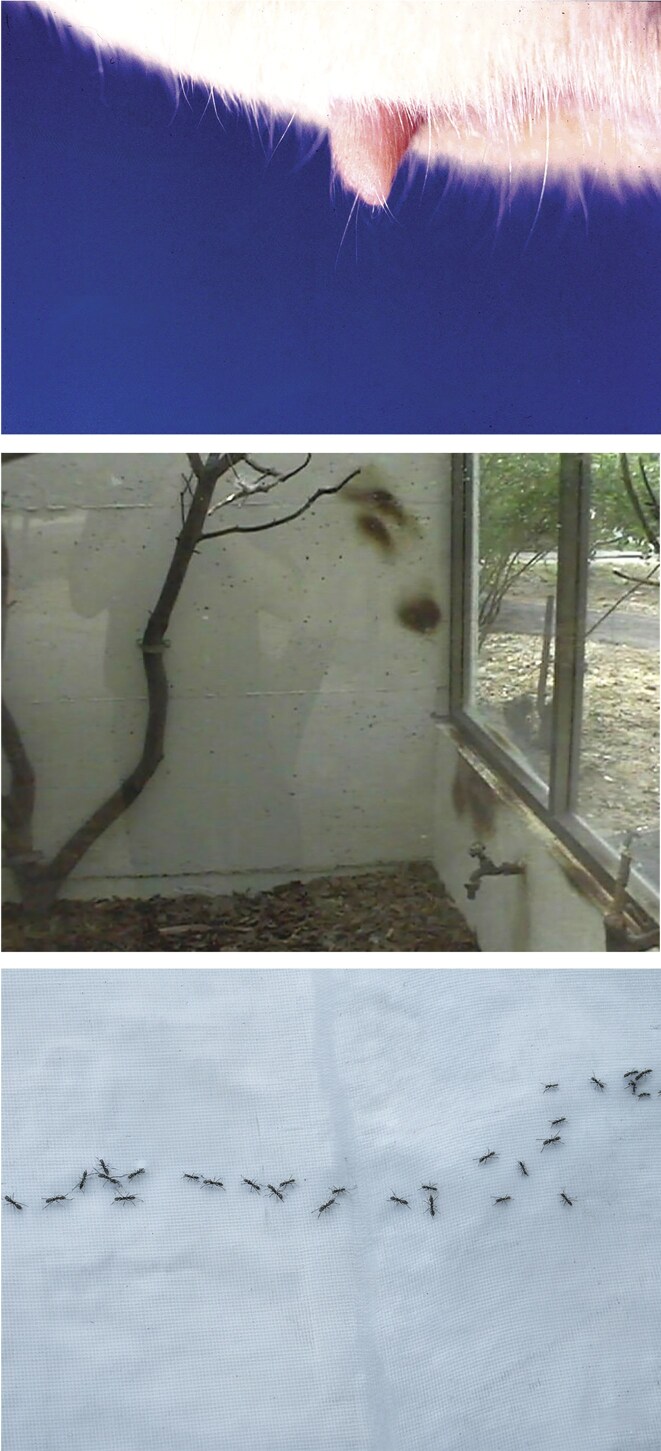
Chemical and physical markers of home boundaries in animals. Long hairs at the tip of the clitoris of a female black rat, facilitating scent marking (top); trail marks like those of many rodent species, in a cage of a Caucasian squirrel (*Sciurus anomalus*; center); and ants follow a pheromone trail exemplifying chemical mapping of space for navigation and colony organization (bottom).

Like reef fish, terrestrial animals often rely on salient landmarks to navigate established paths. For example, striped hyenas (*Hyaena hyaena*) in the Israeli desert frequently travel between prominent boulders or shrubs. In featureless sandy plains, where such landmarks are absent, they instead follow the jeep tracks, which are the only prominent landmark (Bouskilla 1983). This behavior is augmented in captivity, where animals repeatedly deposit substances to demarcate their enclosures, resulting in conspicuous marks, stereotyped paths, and marking behavior at specific locations (Mason 1991). Rodents, which commonly use scent marking to navigate their routes and living range, leave behind dark-brown scent markings that guide their movements when fleeing to shelter. Path marking was particularly noticeable in juvenile black rat (*Rattus rattus*). These rats possess two long hairs at the tip of their penis or clitoris (homologous sex organs, which in rats have a similar external morphology). When placed on an unfamiliar surface, a smear of substance was formed at the tip of the clitoris or penis, and they used these hairs like a brush to paint their trajectory. Ants are renowned for the ability of various species to deposit pheromones (a chemical scent substance), which establish a trail that their nest members rigorously follow. Ants are as sensitive as 1 picogram (10^−12^ gram) per centimeter, and accordingly, only 4 milligrams of trail pheromone are required for 40,000 kilometers of trail that surrounds the globe— amazing indeed (figure 9).

Lorenz noted regular travel along a familiar path in animal behavior and humans, as he described it from his own experience. Driving from home to his office in Vienna before a one-way road plan was enforced, Lorenz realized that he drives one route on the inbound journey and another route on the outbound journey, and this is how he described that: “Rebelling against the creature of habit in myself, I tried using my customary return route for the outward journey and vice versa… the astonishing result was an undeniable feeling of anxiety, so unpleasant that when I came to return I reverted to the habitual route” (Lorenz 1974).

The continuity between animal and human path habits anchored in routine and environmental cues within their living range has prompted researchers to move beyond anecdotal accounts and explore human mobility patterns through large-scale empirical models and tracking technologies. Early models of human mobility patterns were often based on Brownian motion or random walk assumptions, suggesting that human movement consists of a series of stochastic steps in both direction and distance (Camp et al. 2002, Groenevelt et al. 2006, Loannidis and Marbach 2006). However, the availability of large-scale empirical datasets, particularly from Internet activity and mobile phone tracking, has significantly reshaped this view. One early breakthrough came from analyzing banknote tracking data collected via https://www.wheresgeorge.com/, which revealed that human mobility patterns aligns with a Lévy-flight distribution (Brockmann et al. 2006). This indicates that although the travel direction may be random, movement distances follow a power-law distribution: Many short trips are punctuated by occasional long-distance displacements. Mobile phone data further reinforced these findings. A 6-month study of 100,000 users revealed a Lévy-flight pattern and a high degree of temporal and spatial regularity in individual mobility (Gonzalez et al. 2008). These observations were corroborated by GPS tracking (Zhao et al. 2008) and a subsequent study of 50,000 mobile-phone users, which demonstrated that routine patterns such as daily commutes, dominate human mobility, challenging assumptions of spontaneous, unstructured movement (Song et al. 2010).

The above studies show that human movement, like animal behavior, is characterized by frequent returns to key anchor locations (e.g., home, workplace, or school). Remarkably, periodic behavior accounts for 50% to 70% of human movement, whereas social relationships explain only 10% to 30% (Cho et al. 2011). The dominance of regularity was further supported by a study of carpooling in Taiwan, where 73.9% of users followed consistent routes (Chung et al. 2012). This pattern of repetition has been conceptualized in the framework of time geography (Hägerstrand 1970), emphasizing that spatial and temporal routines are structured around fixed anchor locales. Building on this, researchers have explored how human mobility patterns relate to the structure of street networks (Jiang 2009) and found that the hierarchical organization of destinations, individual preferences (Jia et al. 2012), and intervening opportunities (Noulas et al. 2012) shape these patterns. These spatial routines accumulate into broader cognitive and symbolic systems. From daily paths to territorial belonging, we see how home scales-up from personal to planetary.

## East or west, home is best: Scales of home

Although home is usually conceived as an intimate, private space, the notion of home expands far beyond the domestic scale. Across human experience, home operates at multiple nested levels, ranging from the individual dwelling to the neighborhood, the city, the nation, and ultimately the planet. Each scale contributes a distinct layer of meaning, linking personal experience with cultural, symbolic, and even geopolitical belonging. The most prominent symbols of a home extending beyond the front door are hometowns and homelands, both of which are strong representations of a person’s identity. The ancient Greeks suggested that, likewise, animals have a sense of homeland, because they only feel at home in specific places on Earth (Kostuch 2017).

As was detailed before, animals and humans defend and personalize their home and living range to assert identity, as well as securing and optimizing the use of resources. In a similar way, cities and states are a larger-scale spatial frameworks that foster a similar sense of belonging and exclusivity. These geopolitical territories provide the spatial foundation on which collective identity is constructed, reinforced, and symbolically expressed. This territorial grounding enables cities and nations to be emotionally branded: assigned nicknames, emblems, and metaphors that turn abstract spaces into meaningful homes for their inhabitants and diasporic communities. This symbolic framing is evident in how cities and countries acquire emotionally resonant identities: New York as The Big Apple, Paris as The City of Light, Chicago as The Windy City, or Detroit as The Motor City. States and nations similarly adopt emblematic titles: Florida is The Sunshine State, Washington is The Evergreen State, Israel is The Holy Land, China is The Red Dragon, and Japan is The Land of the Rising Sun. These cultural labels function as spatial shorthand, transforming large, abstract geopolitical entities into familiar, identity-rich constructs.

For immigrant and diasporic communities, this layering of home is particularly salient. As we discussed earlier, cultural enclaves such as Chinatowns allow migrants to reestablish symbolic elements of their lost homelands within a foreign environment. These reconstructed spaces offer physical shelter as well as cultural continuity, which represents an echo of the home left behind. At the most expansive scale, the notion of home has been extended to the planet itself. Astronauts frequently describe a shift in consciousness, often referred to as the *o verview effec t*, when viewing Earth from orbit: a sense of emotional attachment to the planet as a shared, fragile, and borderless home (Yaden et al. 2016). In this framing, home becomes planetary: not defined by walls or nationality but by a collective identification with the Earth as the source of life and belonging. From house to habitat, neighborhood to nation, tent to Earth, the meaning of home scales fluidly. It is not confined to a single point in space but instead emerges through layered experiences of orientation, memory, culture, defense, and identity. Across these scales, home remains a central organizing principle in how individuals and communities locate themselves in the world.

## Conclusions

The understanding that most animals are not nomadic and there is an adaptive value to deliberately live and perform day-by-day activities in a confined and familiar space (Stamps 1995) goes back to Darwin, suggesting that “most animals and plants keep to their proper homes, and do not needlessly wander about” (Darwin 1861). We offer in the present article a conceptual synthesis of anthropology, vernacular architecture, environmental psychology, and comparative ethology, thereby constructing a multidimensional framework for understanding home, the most permanent place in life. To complement this conceptual synthesis, supplemental table S1 catalogs the species and behaviors referenced throughout the article, organized by the six dimensions of home. This taxonomy provides the empirical foundation for our framework, demonstrating how diverse ecological and social drivers shape the expression of home across taxa. These examples ground the integrative perspective summarized in table 1, which presents a comparative framework across five key dimensions: functional, spatiobehavioral, affective–symbolic, sociocultural, and temporal–dynamic. These dimensions capture both material and immaterial aspects of home, allowing for cross-species and cross-cultural comparison. Each row highlights how these dimensions are expressed in Western and non-Western human contexts, as well as in the animal kingdom. From shelter construction and spatial organization to emotional attachment, social norms, and temporal cycles, this synthesis illustrates the continuity and variability of home-related behaviors across biological and cultural domains. By framing home as a multidimensional construct, table 1 underscores the central argument of this work: that home is a universal but contextually inflected phenomenon, deeply embedded in ecological logic, cognitive processes, and social meaning. Taken together, these dimensions reveal home not as a static location but as a dynamic construct shaped by biology, culture, cognition, and emotion. Home is a behavioral framework composed of practices, spatial logic, and emotional investment that develop in response to environmental demands and internal psychological needs.

**Table 1.** A comparison of five dimensions of home (left-hand column) in Western humans, non-Western humans, and animals.

Dimension	Description	Western cultures (human)	Non-Western cultures (human)	Animals
Functional	Shelter, safety, rest, reproduction	Apartment, suburban home	Nomadic tent, stilt house	Burrow, den, nest
Spatiobehavioral	Territorial boundaries, zoning, routine movement, home base	House layout (and private rooms), personal boundaries, commuting patterns	Shared courtyards in African compounds, flexible room functions in Japanese homes	Rodent home base, nest zoning, living range, territorial behavior
Affective–symbolic	Emotional meaning, identity, personalization, nostalgia	Teen bedroom, nostalgic childhood home	Shrines and ancestor altars (East Asian homes); symbolic object placement in the yurt	Nest decoration, olfactory marking
Sociocultural	Rules of access, gendered space, hospitality, rituals	Guest etiquette, private property norms, front/back yard use	Tatami room layout (Japan), gendered space separation in Middle Eastern homes	Communal nesting in social voles, pecking distance in birds
Temporal–dynamic	Permanence vs. mobility, transitions, cyclic patterns	Vacation homes, seasonal migration for retirement	Nomadic cycles among Bedouins, Roma and Sámi, transhumance practices	Seasonal migration in quelea birds, coordinated movements in herds

Home also functions as a cognitive and spatial reference locale, a place for orientation, a site of return, and a structure that supports memory and routine. This applies to both humans and animals, as is demonstrated by homing behavior, consistent travel paths, and the use of spatial landmarks. The experience of home persists even in its absence: Individuals without permanent shelter, nomadic groups, and socially organized animals recreate aspects of home through bonding, territorial behavior, and repeated activity patterns. Additionally, we considered how home can be understood at different scales, from the individual and the household to cities, nations, and the planet.

Our synthesis of diverse species indicates that although various nonhuman taxa exhibit traits analogous to specific dimensions of human homeliness, no single species integrates all these dimensions concurrently. This observation is likely a consequence of the distinct evolutionary and ecological pressures acting on different taxa, as well as the inherent complexity and multifaceted nature of the human concept of home. Our analysis further reveals that the human perception and enactment of home is also subject to significant variation across different cultures and life stages. Nevertheless, **t**his does not weaken the current analogy but rather suggests that homeliness is a complex behavior with elements that manifest distinctly, but with conceptual overlap, across both human and nonhuman species. It also underscores that the individual components of homeliness, such as shelter, safety, and social function, are not universally expressed as a single integrated trait but emerge in different contexts and species.

Understanding home as a behavioral and symbolic phenomenon shared across species invites both interdisciplinary interest and critical reflection. By drawing parallels between human and animal spatial behaviors, we challenge the long-standing dichotomy that frames human domesticity as cultural and animal sheltering as instinctive. However, such comparisons inevitably raise questions about anthropomorphism or zoomorphism, and the risks of overinterpreting animal behavior through a human lens. However, we claim that although it is important to avoid projecting complex human emotions or social constructs onto animals, dismissing spatial organization, territoriality, or personalization in animal species as mere automatism is equally reductive. The goal in the present article is not to humanize animals but to approach the concept of home through a broader biological lens, recognizing it as a behavioral pattern shaped by ecological conditions, psychological responses, and social interaction.

This reinterpretation invites a more unified way of thinking across fields that are often treated as separate. It opens possibilities for new insights in areas such as habitat design for animals in captivity, planning and architecture for human environments, conservation efforts, and the development of artificial intelligence systems that must interact with human spaces. It also carries ethical implications. When beings are displaced from familiar environments through imprisonment, homelessness, forced migration, or environmental destruction, the consequences go beyond physical survival. Such disruptions may erode identity, emotional stability, and behavioral patterns that depend on a secure and familiar environment. Our integrated perspective opens new avenues for empirical and theoretical research. Indeed, home is a place of refuge, where one can retreat from the stresses of the outside world and find comfort and peace. It provides a stable base for life and an emotional concept: a feeling of safe havens and belonging, an extension of the individual space, and the core of the living range. Future studies may explore homemaking neural and hormonal substrates, investigate how displacement affects behavioral resilience, or examine the coevolution of spatial cognition and social structure across species. It also invites a rethinking of what it means to feel at home, not just as a sentimental notion but as a shared biological imperative, a behavioral scaffold for navigating the world.

## Supplementary Material

biaf183_Supplemental_Files
