# Supplementary material for: There Is No Place Like Home: Behavioral and Physical Home Traits in Humans and Other Animals
Source: Bioscience. 2025 Dec 4;76(3):222–37. doi: 10.1093/biosci/biaf183 (PMC13032871; doi:10.1093/biosci/biaf183)
Supplement: biaf183_Supplemental_Files [file biaf183_supplemental_files.zip › BIOS.docx]

Efrat Blumenfeld-Lieberthal is Professor at the Azrieli School of Architecture at Tel Aviv University, Israel. Her research lies at the intersection of architecture, urban planning, and spatial analysis, with a particular focus on the dynamics of movement, behavior, and the built environment. She integrates computational modeling, network theory, and behavioral studies to examine how physical space influences human activity and social interaction. Her work bridges disciplinary boundaries between design, spatial cognition, and complex systems, contributing to both theoretical understanding and practical approaches in urban and architectural design.

David Eilam is Professor Emeritus of Zoology at Tel Aviv University, Israel, where he received a career award from the Council for Higher Education in Israel, and currently heads the laboratory for studies of human and animal behavior. His interdisciplinary research is based on fine-grain analysis of behavior, focusing on ritualized and collective behavior, spatial cognition, and threat responses across species—from rodents and owls to humans, including in contexts such as obsessive-compulsive behaviors, sports rituals, and cultural practices. His studies have contributed foundational insights into how motor variability, spatial mapping, and social dynamics shape behavior in both natural and laboratory settings.
