# Supplementary material for: There Is No Place Like Home: Behavioral and Physical Home Traits in Humans and Other Animals
Source: Bioscience. 2025 Dec 4;76(3):222–37. doi: 10.1093/biosci/biaf183 (PMC13032871; doi:10.1093/biosci/biaf183)
Supplement: biaf183_Supplemental_Files [file biaf183_supplemental_files.zip › Table S1.docx]

Table S1: Cross-species expressions of “home” across six dimensions. This table synthesizes examples from the paper, illustrating how diverse taxa express one or more dimensions of “home” — functional spatial organization, personal territory, identity, cognitive/spatial anchoring, scaling, and substitutes for home. For each species, the table highlights observed behavior of different spicies, underlying drivers, relevant references, and their alignment with the six thematic sections explored in the article.

| Taxa / Species | Dimension of “Home” Illustrated | Examples from Paper | Underlying Drivers (“Why”) | References | Thematic Section |
| --- | --- | --- | --- | --- | --- |
| Laboratory rats (*Rattus norvegicus*) | Functional zoning; home-base creation | Partitioning cages into sleep, feeding, and waste areas; establishing home bases in arenas | Hygiene, comfort, spatial efficiency | Leonard & McNaughton 1990; Whishaw et al. 2006 | **Functional Spatial Organization** |
| Voles, jirds, gerbils (*Microtus*, *Meriones*, *Gerbillinae*) | Subterranean zoning; communal use | Burrows with chambers for nesting, food, and escape tunnels | Predator avoidance; caregiving; thermoregulation | Cohen-Shlagman 1981 | **Functional Spatial Organization** |
| Long-eared hedgehog (*Hemiechinus auritus*) | Partitioned space for caregiving | Dens with separate chambers for mother and young | Concurrent caregiving and rest; protection | Mendelssohn & Yom-Tov 1999 | **Functional Spatial Organization** |
| Polar bear (*Ursus maritimus*) | Functional zoning; caregiving | Separate chambers for offspring and mother | Thermal regulation; safe nurturing space | Durner et al. 2003 | **Functional Spatial Organization** |
| Honeybees (*Apis* spp.) | Functional zoning; collective organization | Nurseries, food storage | Efficiency; colony survival | Winston 1991 | **Functional Spatial Organization** |
| Blind mole rat (*Spalax ehrenbergi*) | Solitary territorial burrows | Single burrows spaced apart from others | Resource control; predator avoidance | Cohen-Shlagman 1981 | **Personal Territory** |
| Fat sand rat (*Psammomys obesus*) | Individual burrows with proximity to others | Networks built close to conspecifics but maintained individually | Resource optimization; minimal conflict | Cohen-Shlagman 1981 | **Personal Territory** |
| Gazelles (*Gazella gazella*) | Ritualized territorial marking | Scent marking and spatial rituals around “home ranges” | Territory defense; mating signals | Eilam 2023; Walther 1977 | **Personal Territory** |
| Pronghorn (*Antilocapra americana*) | Territorial marking | Repeated marking and site patrols | Reproductive competition; territory stability | Walther 1977 | **Personal Territory** |
| Antelopes (various spp.) | Spatial regularity; marking | Marking communal areas and movement routes | Group cohesion; signaling | Walther 1977 | **Personal Territory** |
| Golden jackals (*Canis aureus*) | Shared marking; pair bonding | Joint scent-marking of shared spaces | Pair cohesion; defense | - | **Personal Territory** |
| Spotted hyenas (*Crocuta Crocuta*), otters (*Aonyx capensis*),  black rhinos (*Diceros bicornis*),  goitered gazelles (*Gazella subgutturosa)* | Marking and spatial regularity | Use of fixed marking sites | Territory defense; communication | Gorman & Trowbridge 2019; Rowe-Rowe 1992; Schenkel & Schenkel-Hulliger 1969; Blank 2025 | **Personal Territory** |
| Bowerbirds (*Amblyornis inornata*) | Symbolic personalization; identity expression | Decoration of bowers with objects to attract mates | Sexual selection; individual identity | Diamond 1986 | **Home and Identity** |
| White storks (*Ciconia ciconia*) | Place fidelity; identity | Return annually to the same nesting site to breed | Pair-bond reinforcement; cyclical identity | - | **Home and Identity** |
| wandering albatrosses (*Diomedea exulans*) | Spatial fidelity; social continuity | Decades-long loyalty to nest sites, reuniting for breeding | Bonding; continuity of identity despite mobility | Sun et al. 2022 | **Home and Identity** |
| Rock hyraxes (*Procavia capensis*) | Route fidelity; site re-use | Repeated use of specific rocks; predictable movement | Safety; social signaling | Serruya & Eilam 1996 | **Cognitive/Spatial Anchor** |
| Leopards (*Panthera pardus*) | Knowledge of prey patterns; spatial routines | Returning to hunting spots; predictable routes | Energy efficiency; hunting success | - | **Cognitive/Spatial Anchor** |
| Galápagos giant tortoises (*Chelonoidis niger*) | Habitual routes; site fidelity | Returning repeatedly to fixed paths | Energy efficiency; safety | Darwin 1839 | **Cognitive/Spatial Anchor** |
| Social vole (*Microtus socialis*) | Communal nesting; resource sharing | Interconnected burrow networks with shared storage and nursery | Kin cooperation; predator vigilance | Cohen-Shlagman 1981 | **Scaling of Home** |
| African buffalo (*Syncerus caffer*) | Group routines; synchronized movement | Coordinated feeding, resting, and protection | Predator defense; foraging efficiency; social stability | Molszewski 1983; Estes 1991 | **Scaling of Home** |
| Wildebeest (*Connochaetes taurinus*) | Group migration; synchronized behavior | Seasonal migrations; herd resting patterns | Resource tracking; predator defense | Molszewski 1983; Estes 1991 | **Scaling of Home** |
| Impala (*Aepyceros melampus*) | Safety in numbers; social cohesion | Group vigilance; coordinated feeding | Predator detection; social bonding | Elgar 1989; Bednekoff & Lima 1998; Szulkin et al. 2006 | **Scaling of Home** |
| Red-billed quelea (*Quelea quelea*) | Collective mobility; “mobile home” | Huge flocks moving seasonally with food | Safety; resource optimization | Dallimer & Jones 2002 | **Substitutes for Home** |
| Cedar waxwing (*Bombycilla cedrorum*), Black swan (*Cygnus atratus*) | Nomadic flocking | Seasonal movement in large groups | Food availability; safety | Putnam 1949; Kingsford et al. 1999 | **Substitutes for Home** |
| Army ants (*Eciton burchellii*), Migratory locusts (*Locusta migratoria*) | Collective mobile “homes” | Coordinated swarming and resource following | Resource-driven movement; group cohesion | Willson et al. 2011; Lecoq et al. 2022 | **Substitutes for Home** |
